# Benefits for elders with vulnerable health from the Chronic Disease Self-management Program (CDSMP) at short and longer term

**DOI:** 10.1186/s12877-015-0090-4

**Published:** 2015-08-15

**Authors:** Angèle A. G. C. Jonker, Hannie C. Comijs, Kees C. P. M. Knipscheer, Dorly J. H. Deeg

**Affiliations:** Department of Epidemiology & Biostatistics, VU University Medical Center, P.O. Box 7057, 1007 MB Amsterdam, The Netherlands

**Keywords:** Coping, Well-being, CDSMP, Intervention, Frail elderly, RCT

## Abstract

**Background:**

When health declines, older persons may benefit from an intervention program that strengthens their self-management and empowers them to keep in control of their own body and life. Therefore we conducted a Randomized Controlled Trial using the Chronic Disease Self-Management Program (CDSMP) in a sample of 169 older persons in frail health and in need of elderly care.

**Methods:**

We assessed psychological coping resources and wellbeing, pre- and posttreatment and at 6-month follow-up, and investigated whether specific subgroups would benefit in particular from the intervention.

**Results:**

The CDSMP appeared effective with respect to sense of mastery but only in the lower educated participants (p < .05). Furthermore, the intervention stabilized valuation of life in participants, whereas in the controls valuation of life decreased. The high appreciation score and low drop-out are indicative for the applicability of the CDSMP for this specific target group.

**Conclusions:**

We recommend integration of the ingredients of the program into the daily healthcare practice of professionals working with vulnerable older persons. This would involve professional guidance starting from interpersonal equality and emphasising a persons possibilities given their physical or cognitive limitations. This will help older vulnerable persons to focus on their own attainable goals and to experience being successful.

**Trial registration:**

The trial was registered in the Dutch Trial Register as NTR 1173 at 08-03-2008; ‘Is selfmanagement benefical for well-being of average older persons?’ http://www.trialregister.nl/trialreg/admin/rctview.asp?TC=1173

## Background

Because of the increase in life expectancy, a greater number of older people will have age-related diseases and may suffer from the difficulties due to persistent health decline. Studies using different frailty-instruments showed prevalence rates between 12 % and 36 % in people aged 65 and over in the Netherlands [1, 2]. Lee et al. [3] state that prevalence of frailty increases with age, affecting an estimated 16 % of those aged 80 to 84 and 26 % of those aged 85 and older. Although the Dutch disability level is among the lowest in developed countries [4, 5] a considerable proportion of the population still will have to face the challenge of coping with health decline. Older persons confronted with deteriorating health often experience lower levels of well-being [6–8]. The availability of coping resources like mastery, self-esteem and self-efficacy have been shown to buffer the negative influence of deteriorating health on well-being; moreover, associations between persistent health decline and decreasing well-being are partly explained by decreasing availability of psychological coping resources [9–12].

In order to optimize well-being of the growing number of older persons in vulnerable health, it seems a priority to enhance coping resources, and by doing so, to empower older persons. Therefore it is important to investigate specific interventions that are developed to maintain or improve optimal coping resources in these persons. Self-management programs are proposed as one of the ways for older persons to more actively manage their own process of ageing in such a way that the availability of coping resources and, as a consequence, well-being is increased and maintained as long as possible [13]. Specifically the Chronic Disease Self-Management Program (CDSMP) is a structured intervention that emphasizes the strengthening of self-management by older persons with deteriorating health in order to empower them to keep in control of their own body and life [12, 14–17].

The CDSMP is the only intervention programme that focuses on people with one or more chronic diseases regardless of the specific disease, and that aims at stimulating patients to become more actively involved in the management of their own health and enabling them to take better care of themselves [18]. Previously, we conducted a systematic review [19] on nine Randomized Controlled Trials of the CDSMP. Overall, the studies reviewed showed that the CDSMP led to more physical exercise, less health distress, better self-care and had a beneficial effect on self-efficacy measures. Thus, the CDSMP seems a promising intervention. However, in most RCT’s the average age was not very high. So far, the effectiveness of the CDSMP has not yet been determined in frail older people with heterogeneous chronic diseases and who are dependent on old-age care. Therefore, we conducted an intervention study on the CDSMP in this population. It is hypothesised that participating in the CDSMP leads to improved coping resources and well-being.

A second aim of our study was to investigate whether specific subgroups benefit more from the intervention than others. For instance, it may be expected that persons with good cognition and higher education benefit more than persons with low cognition or education. Findings may result in a specific profile of people most likely to benefit from the program.

## Method

### The intervention

The central aim of the Chronic Disease Self-Management Program (CDSMP) is to teach people to cope with multiple chronic diseases. The CDSMP is based on prior experience with an arthritis self-management program, literature review, needs assessments and the theoretical framework of self-efficacy [9, 15, 20]. The underlying mechanism that explains the positive effects on health behaviour, health status, self-management behaviour and health care utilization, is assumed to be self-efficacy. This is defined as ‘believing in one’s capability to organize and execute the courses of action required to produce given attainments’ [20]. The CDSMP incorporates strategies to enhance self-efficacy and by doing so to enhance self-management behaviour and health related outcomes. By means of weekly action-planning and feedback, participants modelling behaviour and problem-solving for each other, re-interpretation of symptoms, group problem solving and individual decision-making, the program is executed [21]. Three principal assumptions underlie the CDSMP:

1. People with different chronic diseases have similar self-management problems and disease-related tasks.
2. People can learn to take responsibility for the day-to-day management of their diseases.
3. Confident, knowledgeable patients practicing self-management will experience improved health status and will utilize fewer health care resources.

The program is accessible and easy to implement, because it is inexpensive and widely available, and the intervention can be delivered by trained lay-persons. The CDSMP focuses on several topics including physical exercise, nutrition, breathing, emotions, communication and medication, which are discussed during six weekly sessions of 2.5 h.

The study protocol has been approved by the Ethical Review Board of the University Medical Center Groningen (UMCG). The study protocol can be obtained from the corresponding author.

### Setting and study sample

We recruited older people who participated for one or more days a week in an elderly day-care facility with several locations. The research team advertised the intervention through personal announcements to participants and information sessions by visiting the day-care facility themselves. Formal caregivers at the facilities were informed and potential participants were sent an information letter by their caregivers, 169 of whom gave written informed consent. With the remaining 21 persons, no contact could be established. Research assistants then contacted the participants and carried out a baseline measurement.

We aimed for a power of 80 % to detect a minimum difference in the main outcome measures between two independent sample means, at alpha .05 [22]. Power analysis revealed that we needed to include 160 participants.

### Randomisation

Participants were randomised to the CDSMP programme or to a waiting list control group that received care as usual and was promised participation after 6 months. Randomisation was based on the existing units of day-care groups in two day-care locations. Thus, at each location of the day-care facility, candidate participants were randomised groupwise per weekday on which they normally received the day-care. Groups of 10–15 participants were included. Each group was supervised by two well-trained nurses, to guarantee continuation of the group sessions in case of absence of one of the supervisors.

### Design

Baseline measurements included participant characteristics and initial values of the outcome measures. After the intervention of 6 weekly sessions were completed, follow-up measurements of the outcome measures was conducted. These measurements were repeated 6 months after completion of the intervention.

### Measurements

#### Outcome measures

The choice for coping and wellbeing as outcome measures was based on the frequently reported evidence that psychological coping resources, such as mastery [23], self-esteem [24] and self-efficacy [9] favorably affect a person’s way of coping with deteriorating health [12, 25]. The main outcome measures are psychological coping resources (mastery, self-esteem and self-efficacy) and wellbeing (positive affect, life satisfaction, valuation of life and depressive symptoms).

Sense of mastery is conceptualised as the extent to which a person perceives him or herself to be in control of events and ongoing situations and reflects the perception of the ability to manage them. This was measured by a 5-item abbreviated version of the Pearlin Mastery scale [11, 26] which included questions like ‘I have little control over things that happen to me’. Each item is scored on a five-point scale, the total score is the sum of the ratings, with range 5–25, such that a higher rating indicates more feelings of mastery.

Self-esteem is measured by a scale that consists of four questions like, ‘feeling self-assured’, ‘positive attitude towards one’s self’and ‘feeling useless’ that are scored on a five-point scale [11, 27]. The score is the sum of the ratings, with range 4–20. People with higher self-esteem (i.e., higher scores) are supposed to have a more positive view of their identity.

Self-efficacy refers to personal judgements of how well behavior can be implemented in situations that contain novel, unpredictable or stressful elements as well as ordinary situations [9]. Self-efficacy was measured by a twelve-item version of the Perceived Self-Efficacy Scale [28, 29]. The scale included questions like ‘If I made a decision to do something, I will do it.’ and ‘I have difficulties solving problems well in my life’. Each question is scored on a five-point scale, the total score is the sum of the ratings, with range 12–60, with a higher score indicating a higher level of self-efficacy.

Depressive symptoms were measured with the Centre for Epidemiological Studies-Depression scale (CES-D) [30]), which assesses depressive symptoms. The CES-D is a 20-item scale that asks participants to indicate how frequently they experienced certain psychological symptoms or feelings during the previous week. Each question is scored on a four-point scale, the total score is the sum of the ratings, with range 0–60, with a higher score indicating more depressive feelings.

Positive affect was measured using a subscale of the CES-D. Radloff [30] described four separate dimensions of the CES-D. One of the dimensions is positive affect, including four of the CES-D items which refer to positive feelings: ‘enjoying life’, ‘feeling happy’, ‘being hopeful about the future’ and ‘feeling as good as other people’. The items are scored on a four-point scale. This sub-scale ranges from 0 (low) to 12 (high). The use of this subscale as an independent concept is supported by previous research [31]. Higher scores indicate higher positive affect.

To assess Life satisfaction, two questions ‘Have you been satisfied with your life lately?’ and ‘Are you satisfied with your life, up until now?’ were asked [32] The questions are scored on a five-point scale, and the sum score ranged from 2 (very dissatisfied) to 10 (very satisfied).

Valuation of Life (VOL) is considered as a cognitive scheme which refers to “the subjective experienced worth of a person’s life, weighted by the multitude of positive and negative features whose locus may be either within the person or in the environment” [33]. The Dutch version of the VOL-scale [34] consists of 12 statements, about the value of life, such as:’It is difficult for me to find meaning in my daily routine’ or ‘At this moment I have a strong will to live’. Each item is scored on a five-point scale, with the sum score ranging from 12–60, higher scores indicate higher valuation of life.

#### Potential confounders

Variables that may confound the effect of participating in the CDSMP on the outcome measures were taken into account. Age, sex, income category, partner status, years of education, help received with personal care and household tasks, chronic diseases, and cognitive function were considered potential confounders. Age was measured by years and months of age and sex was measured by observing the gender (male or female). Income category was measured by asking about income with three questions on receiving state pension, private pension, and savings. Partner status was measured by asking whether the respondent was living with someone they considered as their partner (yes/no). Education was measured by asking about the number of years of education that was received. Personal care and household care were measured by asking whether the respondent received help with personal and household care, respectively (yes/no).

The presence of chronic diseases was determined by asking the respondents whether they had any of the following diseases: cardiac disease; peripheral arteriosclerosis of the abdominal aorta or the arteries of the lower limb; stroke; diabetes mellitus; lung disease (asthma or chronic obstructive pulmonary disease); cancer; arthritis; or any other major chronic disease. The number of chronic diseases was calculated by summing all the specific diseases reported. In a validation study, the respondents’ self-reports were compared with information obtained from their general practitioners, and were found to be sufficiently reliable [35]. Cognitive functioning was measured by means of the Mini Mental State Examination (MMSE) [36] a frequently used screening instrument for global cognitive dysfunctioning. For 23 questions and tasks the respondents scored 1 or more points if they gave the correct answer or performed the task correctly. The scores could vary between 0 (all answers incorrect) and 30 (all answers correct). Higher scores indicate better cognitive functioning.

### Statistical analyses

First, unpaired t-tests and chi-square tests were performed to compare the participant characteristics and the baseline scores of the intervention and the control group regarding coping and well-being outcomes. Next, paired t-tests and repeated measures-analyses using General Linear Models were performed to assess treatment effects between baseline and post-intervention and between baseline and 6-month follow-up. Differences and changes were considered significant when p < .05.

To examine whether the participant characteristics moderate the association between the intervention and well-being or coping resources, a series of multivariate analyses using General Linear Models was performed for each characteristic. Each multivariate model examined whether the product term (intervention X characteristic), was significant (p < .10). When the interaction term was found significant, the effect of the intervention was investigated in stratified analyses. We stratified on quartiles and median or mean of the characteristic to investigate the optimal distinction between groups.

## Results

### Subjects

**Fig. 1:**
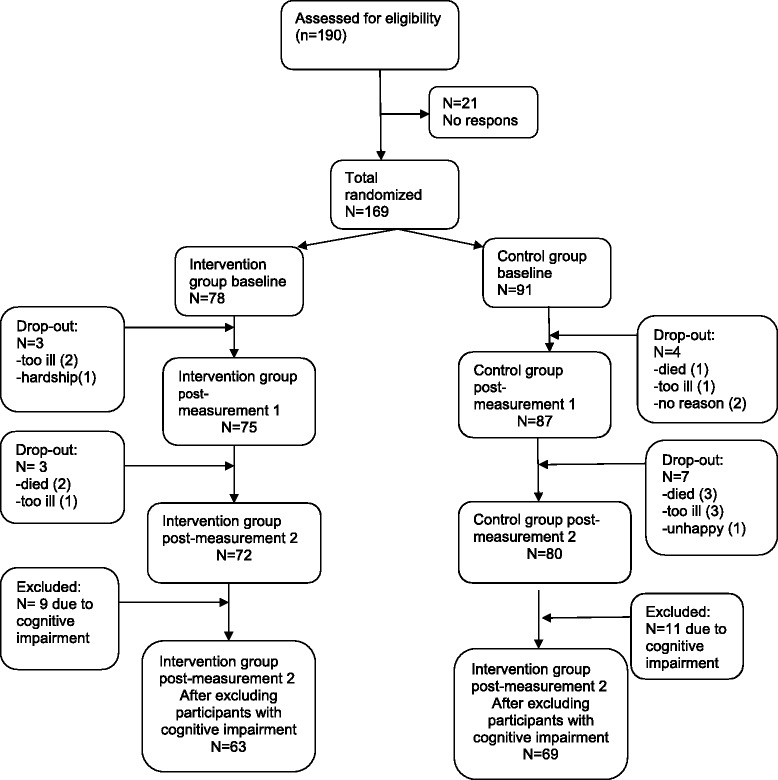
Enrolment procedure (original N = 190)

Of the169 participants, 78 (46 %), aged 81 years, were assigned to one of the intervention groups, while the rest, aged 83 years, constituted the control groups. In Fig. 1 the inclusion and drop-out of participants is shown. As can be seen, no patients dropped out before starting the intervention. Seven participants did not complete the first post-intervention interview because they were (too) ill (N = 3), died (N = 1), or were confronted with hardship in the family (N = 1). Two participants did not give a specific reason for quitting. From these seven drop-outs, three had been assigned to the intervention group. Another 10 persons did not complete the 6-month follow-up interview due to illness (N = 4), death (N = 5) and one person from the control group stopped participation because she was unhappy waiting for the course, leaving 152 participants in the study (72 in the intervention group and 80 in the control group).

The intervention took place in existing groups in an elderly day-care facility. However, within these groups some participants had severe cognitive impairment at baseline or follow-up (MMSE ≤15 [37]. In order not to compromise the group dynamics and hurt people’s personal feelings, we chose to treat these participants like the others considering the follow-up interviews and actual participation in the intervention. However, the participants with severe cognitive impairment were excluded from the analyses. Therefore our study sample consisted of 132 persons, 63 of whom participated in the CDSMP and 69 were in the waitinglist control group. Attendance of the intervention meetings was high with an average of five of the six sessions that were offered.

Characteristics of participants at baseline are shown in Table 1. At baseline, the intervention group did not differ from the control group with respect to age, sex, income, partner status, help with personal care, household care, chronic diseases and cognitive functioning. Education level was somewhat lower in the intervention group (p = 0.08). Scores on Self-efficacy, Mastery and Valuation of Life were significantly lower in the intervention group than in the control group.

**Table 1:** Characteristics of the sample at baseline

	Intervention group	Control group	P-value*
	*N* = 63	*N* = 69	
A. Participant characteristics			
Sex, % female (SD)	90.5 (.30)	85.5 (.36)	.38
Age, mean (SD)	81.57 (7.53)	83.09 (5.75)	.20
Education in years, mean (SD)	8.46 (2.54)	9.32 (3.06)	.08
Chronic diseases, mean (SD)	1.97 (1.38)	2.23 (1.29)	.26
Frailty, mean (SD)	5.45 (2.90)	4.91 (2.75)	.28
Cognitive functioning, mean (SD)	24.52 (4.12)	25.62 (3.38)	.10
B. Coping resources and Well-being			
Self-efficacy, mean (SD)	39.56 (6.22)	41.77 (6.22)	.04
Mastery, mean (SD)	22.02 (4.98)	24.41 (4.84)	.01
Self-esteem, mean (SD)	9.98 (3.02)	10.55 (2.47)	.24
Life satisfaction, mean (SD)	7.79 (1.30)	7.72 (1.32)	.55
Valuation of Life, mean (SD)	41.38 (6.83)	44.46 (6.23)	.01
Positive affect, mean (SD)	11.38 (3.19)	11.90 (2.85)	.33
Depression, mean (SD)	34.05 (9.27)	32.84 (8.25)	.43

* t-tests and chi square test

### Effect of the intervention

For each group, paired t-tests were performed between baseline and post-intervention and between baseline and 6-month follow-up (Table 2). The results from the post-intervention 6-week assessment, show that the outcome measures Self-efficacy and Valuation of Life decreased significantly (p < .01) for the control group whereas they stayed stable in the intervention group. At 6-month follow-up, scores on Self-efficacy (p = .01) and Valuation of Life were still lower in the control group (p = .02). Furthermore, on Mastery and Depression results showed positive changes in the intervention group at 6-month follow-up. Mastery improved (p = .01) whereas scores on Depression decreased (p = .05) significantly. Self esteem, Positive Affect and Life satisfaction did not show any difference between the control and intervention group, at both follow-ups.

**Table 2:** Differences between the intervention group and the control group with respect to the outcome measures

	Baseline - post intervention				Baseline - 6 month follow up			
	M	t	p	sd	M	t	p	sd
	Control group							
Self-efficacy	−2.43	−3.83	<.001	5.19	−1.74	−2.64	<.01	5.47
Mastery	-.40	-.82	.41	4.02	-.61	-.99	.32	5.09
Self esteem	-.27	-.97	.34	2.27	.43	.15	.28	2.39
Depression*					1.10	.94	.35	9.78
Positive affect	.15	.37	.71	3.29	-.45	−1.13	.26	3.29
Life satisfaction	.06	.39	.70	1.25	-.09	-.59	.56	1.23
Valuation of Life	−1.93	−3.03	<.001	5.21	−1.61	−2.50	<.02	5.35
	Intervention group							
Self-efficacy	-.10	-.15	.88	5.05	-.48	-.71	.48	5.36
Mastery	.06	.12	.90	4.14	1.49	2.81	<.01	4.22
Self esteem	-.33	−1.21	.23	2.19	.46	1.39	.17	2.62
Depression*					−1.7	−2.01	<.05	6.71
Positive affect	-.38	−1.00	.32	3.03	.10	.23	.82	3.25
Life satisfaction	-.13	-.79	.43	1.28	-.05	-.28	.78	1.34
Valuation of Life	−1.08	−1.41	.16	6.1	-.67	-.86	.39	6.14

*Note: Full CES-D measured only at baseline and 6-months follow-up

In addition, we conducted analyses of variance by means of repeated measures to assess treatment effects, adjusted for confounders and the baseline scores of the outcome measures. Table 3 shows the results of the effect of participating in the CDSMP on change in coping resources and well-being at short (6 weeks) and longer (6 months) term. We included years of education as the only potential confounder because a difference between both study groups was observed at baseline (p .08). A significant effect of participating in the CDSMP was found for Mastery and Valuation of Life. Compared to the control group, participating in the CDSMP led to significantly higher scores on Mastery at short-term. However, the effect size was rather small. Valuation of Life was stable for CDSMP participants immediately after the course and this effect was still present at six months, whereas participants of the control group were confronted with decreasing scores. These effects were also small (partial eta^2^ < 0.06). Participating in the CDSMP did not lead to change in the other outcome measures.

**Table 3:** Longitudinal association between participating CDSMP and change in coping resources and well-being (adjusted for years of education)

	Self efficacy		Mastery		Self esteem		Valuation of Life		Positive affect		Life satisfaction		Depression	
	T0-T1	T0-T2	T0-T1	T0-T2	T0-T1	T0-T2	T0-T1	T0-T2	T0-T1	T0-T2	T0-T1	T0-T2	T0-T1	T0-T2
Multivariate model														
F	.55	1.59	6.04	2.76	1.50	.52	4.37	5.33	2.4	.12	.77	.13	n.a.	.011
P value	.46	.21	.02	.10	.22	.47	.04	.02	.12	.73	.38	.72		.74
Partial Eta2	.00	.01	.05	.02	.01	.00	.03	.04	.02	.00	.01	.00		.00

### Specific subgroups

For investigating a moderator effect of participant characteristics on the effect of the intervention on the outcome measures after 6 months, the product term of intervention X characteristic was entered into the separate models for each characteristic. The product term intervention X education was significant with mastery as the outcome. The product terms intervention X education and intervention X cognitive functioning were significant for depressive symptoms as the outcome (Table 4).

**Table 4:** Interaction analysis of the effect of potential predictor on outcome measures (only those outcome measures with significant interactions are shown, *P* < 0.1)

Product term	Outcome	F	P	Partial Eta2
Intervention X				
Age	Depression	.03	.87	.00
Sex		.03	.87	.00
Education		3.49	.06	.03
Cognition		3.00	.09	.02
Frailty		1.76	.19	.01
Age	Mastery	.13	.72	.00
Sex		.05	.83	.00
Education		2.88	.09	.02
Cognition		.10	.75	.00
Frailty		.48	.49	.00

For the outcome mastery, the optimal cut-off in years of education was < = 9 years and >9 years of education: each of which constituted 50 % of the sample. Multivariate analyses of variance showed a significant positive effect (p < .05) of intervention on mastery for respondents with low education. This was in contrast with the results for respondents with higher education, who showed no significant effect from the intervention (Table 5).

**Table 5:** Stratified analysis on cognitive functioning on the effect of PDF on change in Depression and on educational level on the effect of PDF on change in Mastery and Depression

Change in Depression							
	F	P	Partial Eta2	Mean scores			
				Control		Intervention	
				T0	T2	T0	T2
Cognition Low (MMSE < =25)	1.99	.16	.03	32.2	32.3	36.6	34.1
Cognition High (MMSE > =26)	2.89	.09	.04	33.3	35.0	31.4	30.5
Change in Mastery							
	F	P	Partial Eta2	Mean scores			
				Control		Intervention	
				T0	T2	T0	T2
Education Low (<=8 years)	4.19	<.05	.06	25.1	23.1	21.3	23.3
Education High (> = 9 years)	.42	.52	.01	23.8	24.4	22.9	24.0
Change in Depression							
	F	P	Partial Eta2	Mean scores			
				Control		Intervention	
				T0	T2	T0	T2
Education Low (<=8 years)	.87	.35	.01	32.2	33.1	35.3	33.5
Education High (> = 9 years)	1.231	.27	.02	33.4	34.7	32.4	30.8

With respect to the outcome cognitive functioning after 6 months, stratification based on median scores of respondents (MMSE < =25 & MMSE >25) showed a trend (P = .09) that persons with better cognitive performance seemed to benefit from the intervention (Table 5). This was in contrast to the results for respondents with lower cognitive performance, who showed a non-significant increase of depressive symptoms 6 months after the intervention. Stratification of the sample based on the low and high quartiles of the MMSE did not show significant effects from the intervention in the distinguished groups.

Finally, for the outcome depressive symptoms after 6 months, stratification of the sample for the level of education according to the median and the lowest and highest quartiles, did not result in significant effects from the intervention although the depressive symptoms decreased in the intervention group whereas persons in the controlgroup showed an increase of depressive symptoms.

### Subjective evaluation of participating in the CDSMP

Table 6 shows the results from the subjective evaluation of the intervention among all 63 participants. The participants attended on average 5.7 of the 6 sessions that were provided. They rated the CDSMP with an appreciation of 8.0 (scale 0–10). All of them enjoyed following the CDSMP and 92 % claimed usefulness of the content. Almost all participants (98 %) found the way the intervention was presented agreeable.

**Table 6:** Qualitative evaluation after participation CDSMP

Participants (*N* = 63)	Enjoyable	Usefulness	Teaching approach	Attendance	Judgement on appreciation
	(%)	(%)	(%)	(6 sessions)	(0–10)
Totally agree	73	57	87	5.7	8.0
Agree	17	35	11		
Neutral	0	5	2		
Disagree	0	2	0		
Totally disagree	0	2	0		

## Discussion

Our study of the Chronic Disease Selfmanagement Program in elders with a vulnerable health, with a mean age of around 81 years showed that the program seems effective with respect to sense of mastery but only in the lower educated participants. Also, the intervention had a stabilizing effect on valuation of life, whereas in the controls valuation of life decreased. Furthermore, almost all of the participants scored positively on the content and style of the program, gave high appreciative scores, showed a high attendance rate and low drop-out. In all, our findings support the applicability of the CDSMP for this specific target group.

Thus far, no other RCT of the CDSMP included Mastery as an outcome variable, although mastery has been shown to be important for maintaining well-being in people with deteriorating health. It has frequently been reported that psychological coping resources, such as mastery, favorably affect a person’s way of coping with deteriorating health [25, 38]. It has also been found that greater availability of coping resources is associated with better well-being in chronically diseased persons [39–42]. In addition, a mediating and moderating effect of mastery was demonstrated in our previous study on the association of deteriorating health with wellbeing [19].

In addition to mastery, well-being is also under pressure from deteriorating health [8]. Research in older persons confronted with deteriorating health shows that various aspects of well-being decrease (e.g. [6, 7, 43]). The results of our well-being measures positive affect and life satisfaction did not show an improvement, but we did find a positive effect on Valuation of Life. From an earlier review [19] we learned that the CDSMP was consistently beneficial for Health behaviour, especially with regard to the variables of exercise and self-care. For Health status, the majority of studies only showed improvement in the domain of health distress. Most of the studies that investigated self-efficacy showed convincing improvement in self-efficacy, cognitive symptom-management and mental stress-management.

From this study on nine RCTs using the CDSMP, only Haas [44] found an improvement on emotional well-being among their sample of older adults with chronic low back pain. Griffith [45] and Kennedy [46] showed improvement in psychological well-being and quality of life in younger persons with co-morbidity.

As the program includes action plans that are formulated after each of the sessions and evaluated at the start of the next session, one might expect that the CDSMP would lead to better self-efficacy scores. This has indeed been reported in several studies [45–49] in samples with various chronic diseases. However, in our sample of older people in vulnerable health we did not find an effect on self-efficacy. In a Dutch sample of chronically diseased patients [18] did not find an effect either. They argued that this might have been caused by a ceiling-effect because their patients already had high baseline levels. Our more vulnerable population could have improved in self-efficacy, because they initially scored well below the ceiling of the scale. In fact, self-efficacy scores declined in both intervention and control groups, but less so in the intervention group. Possibly, a longer follow-up would have yielded significant differences.

In addition to the evidence of an overall benefit from participating in the CDSMP in frail older adults, on Mastery directly after the intervention and on Valuation of Life after 6 months, we also found that low educational level and good cognitive functioning increase the likelihood of profiting from the program on coping and well-being outcomes. A low educational level may imply more room for improvement and development in the participants. Improvement of mastery for lower educated older persons in vulnerable health seems of great importance considering several studies that show low educational level to be associated with a lower sense of mastery (e.g. [24, 38]) and that low mastery has been shown to increase the risk of poor mental health [50]. In the study of Dalgard et al. [51] a sense of mastery even emerged as a strong mediating variable between level of education and psychological distress.

The observed beneficial effect of the CDSMP for frail persons with good cognitive functioning on depressive symptoms seems also relevant, considering the high prevalence of depression among older people [52].

### Strengths and limitations

We consider it a strength that we conducted, to our knowledge, the only RCT of the CDSMP that was performed with this specific target group of very old and frail persons with healthcare needs. A further strength is that we were able to conduct a randomized controlled trial among these vulnerable older persons. A host of intervention studies on CDSMP were conducted with only a pre-posttest design [19] whereas only well-designed RCTs can help us to understand what type of intervention promotes a specific change in behaviour, because testing of interventions is possible only when a well-chosen and well-described control group is in place. If not, this may lead to “evidence inspired” rather than evidence based practice [53].

A limitation of our study concerns the number of participants. To perform further in-depth analyses on the original sample – that already was rather small – the number of respondents that we could include was sometimes low. This reduces the power of our study, and may have led to an underestimation of the effects of CDSMP. We aimed for a power of 80 % but due to drop-out, the needed sample size of N = 160 was not achieved.

We feel that our vulnerable participants could have benefitted more from the program had it included more sessions to keep the self-management attitude under attention.

A further limitation is the application of multiple comparisons. Concerning this issue, we have not made a Bonferroni correction because of the chance that we have found a type two error is very small (one of 20) and the findings in our study are not unexpected and consistent with other research [54].

## Conclusion

Because vulnerable older persons, who are often confronted with deteriorating health, may benefit from adequate coping strategies, we consider this program to be successful because of its positive effects on mastery and valuation of life. Also the fact that almost all of the participating persons scored positively on the content and style of the program, the high attendance rate, and the low drop-out is indicative for the applicability for this specific target group. When older people’s health deteriorates to a certain point, participating in a course will become difficult. We therefore recommend integration of the ingredients of the program into the daily healthcare practice of professionals working with vulnerable older persons. This may be achieved when professional guidance starts from people’s possibilities, considering their physical or cognitive limitations, stimulating them to focus on their own attainable goals and providing them with the experience of being successful. Our findings suggest that integrating ingredients of the program into daily healthcare practice might be beneficial for frail older persons in institutions. However, implementation studies are necessary to study this further.
